# Action observation and imitation: Corticospinal responses and hemispheric dominance

**DOI:** 10.1162/imag_a_00419

**Published:** 2025-01-03

**Authors:** Niloufaralsadat Hashemi, Tom Chau, Deryk S. Beal

**Affiliations:** Bloorview Research Institute, Holland Bloorview Kid’s Rehabilitation Hospital, Toronto, Ontario, Canada; Institute of Biomedical Engineering, University of Toronto, Toronto, Ontario, Canada; Department of Speech-Language Pathology, Temerty Faculty of Medicine, University of Toronto, Toronto, Ontario, Canada; Rehabilitation Sciences Institute, Temerty Faculty of Medicine, University of Toronto, Ontario, Canada

**Keywords:** transcranial magnetic stimulation, corticospinal excitability, action observation, action imitation, mirror neuron system, cerebral dominance

## Abstract

The response of the motor neuron system to the observation of lateralized movements and expectation mismatches remains elusive. We investigated how observation of hand movement modulates corticospinal excitability as measured by motor evoked potentials to single pulse transcranial magnetic stimulation (TMS). Twenty-seven adults watched a series of egocentric video recordings of hands, with one hand either squeezing a foam ball as expected or both hands remaining stationary. Task conditions comprised observation-only and observation with synchronous imitation of the depicted motor action. Single TMS pulses were delivered to the motor cortex contralateral to the dominant hand at the video frame of maximal hand closure for squeeze videos and randomly for no-squeeze videos (in both observation-only or observation with synchronous imitation). We analyzed MEPs, from the First Dorsal Interosseous (FDI) muscle of the dominant hand. Observation alone (absence of motor intention) did not enhance corticospinal excitability; however, when paired with imitation (presence of motor intention), it tended to increase MEP amplitudes, regardless of the attended side (dominant or non-dominant) or depicted action (squeeze or not). Among conditions in which the dominant hand remained stationary, MEP amplitudes were elevated (p = 0.004) in observation with imitation of non-dominant hand squeezing, suggesting hemispheric dominance in coordinating motor actions. Additionally, MEP latencies tended to decrease during synchronous imitation of squeeze videos. Our findings support the consideration of observation with synchronous imitation as a task for brain state-dependent brain stimulation protocols for optimizing neuromotor recovery.

## 1 Introduction

Motor resonance, driven by the mirror neuron system (MNS), activates the motor system upon observing actions (Rizzolatti & Craighero, 2004;Sartori et al., 2014). Motor resonance underpins observational therapeutic strategies, which hold promise for upper-limb rehabilitation in conditions like stroke, Parkinson’s disease, and cerebral palsy, where impairments of the upper-limb adversely affect function and social participation (Stolbkov & Gerasimenko, 2021;Tani et al., 2018). Upper-limb observation and imitation have thus become key to understanding and leveraging motor resonance for therapeutic advancement.

Single-pulse Transcranial Magnetic Stimulation (spTMS) is used to probe corticospinal excitability with excellent temporal precision, triggering motor-evoked potentials (MEPs) commonly in hand muscles. These MEPs, measurable through electromyography (EMG), serve as an index of corticospinal excitability strength. Hence spTMS has been widely used to study neural excitability during action observation, providing insights into motor resonance and illustrating how observation of actions is converted into measurable neural responses (Naish et al., 2014).

TMS may be used offline or online to investigate action observation. Offline protocols administer stimulation before and after participants are exposed to an action observation, crucial for assessing long-term neural impact. Conversely, online protocols examine real-time neural changes during observation, applying stimulation at key moments to capture immediate responses to observed movements, offering insights into motor resonance dynamics. This study focuses on an online TMS protocol.

Online TMS protocols contribute to the understanding of the immediate effects of action observation on corticospinal excitability, elucidating three aspects of the mirror neuron response (Naish et al., 2014): (1) muscle-specific engagement, (2) the direction (increase or decrease) of neural excitation, and (3) the precise timing of modulation prompted by the observed action.

Muscle specificity in the MNS results in selective motor pathway activation, mirroring observed actions (Borroni & Baldissera, 2008;Fadiga et al., 1995). Studies show this specificity in response to action observation is influenced by postural congruence (Cardellicchio et al., 2020;Urgesi et al., 2006) and can modulate motor inhibition and interhemispheric inhibition (IHI) (Cardellicchio et al., 2020;Tian et al., 2021). The MNS is sensitive to hand dominance and hemisphere-specific sensory-motor integration (Aziz-Zadeh et al., 2002). The MNS’s muscle specific response is independent of the observer’s familiarity with the performing agent (Maeda et al., 2002) and accommodates the encoding of physically unfeasible movements (Romani et al., 2005). These studies highlight the MNS’s complex capacity for muscle specificity.

Online TMS studies reveal that the magnitude of neural modulation in response to action observation varies with the observer’s intention (Hardwick et al., 2012), anticipation of action (de Beukelaar et al., 2016), and the nature of the action. In particular, neural modulation is more pronounced for transitive (involving objects) versus intransitive actions (without objects or specific goals) (Enticott et al., 2010;Maeda et al., 2002). Enhanced corticospinal excitability has been observed in response to dual-handed movements (Cracco et al., 2016), social interactions (Aihara et al., 2015), and emotionally charged scenarios (Enticott et al., 2011), with direct eye gaze further augmenting this effect (Prinsen et al., 2017;Prinsen & Alaerts, 2020). These studies demonstrate the sensitivity of the MNS to a complex array of stimuli.

Naish et al. (2014)highlighted the importance of pinpointing the timing of corticospinal excitability changes during action observation, with early modulations starting as soon as 75 ms after action onset, indicating swift MNS engagement.Gangitano et al. (2001,^2004^) found peak TMS-induced MEP amplitudes closely tied to the instant of maximum finger aperture during the observation of reaching-grasping tasks. This finding confirms the temporal precision with which the MNS matches neural excitation with key action phases.

Several gaps in the literature persist.Tian et al. (2021)andAziz-Zadeh et al. (2002)only considered unilateral (single-hand) observations. The MNS response to lateralized cues (the observation of left versus right-hand movements) remains unknown. Likewise, the corticospinal excitability of the dominant hand during imitation of non-dominant hand movements remains unexplored. Addressing these gaps would elucidate lateralized effects on corticospinal excitability and interhemispheric interaction.Gangitano et al. (2004)highlighted the need for congruence between observed actions and expected movement patterns, yet little is known about the effects of expectation mismatches concerning the outcome of the observed action on corticospinal excitability. In summary, we have limited understanding of the MNS during lateralized movements and expectation mismatches. Addressing these gaps will contribute to refining observational rehabilitation strategies.

Our study investigated corticospinal excitability during hand movement observation and imitation, with a specific focus on the dominant hemisphere’s response to the movement of the adjacent left or right hand, each preceded by a laterally positioned cue to incite anticipation. This study had two primary hypotheses:

1. Observation-induced vs. imitation-induced modulation: Corticospinal excitability is enhanced by both observing and imitating hand squeeze actions, relative to no-squeeze actions, mirroring each other’s effects but with varying magnitudes.
2. Influence of movement laterality: The laterality of the observed action (i.e., a left- or right-hand squeeze) will uniquely influence excitability in the participant’s dominant hemisphere.

Our approach addresses the highlighted gaps and advances our understanding of motor resonance and therapeutic potential of the MNS.

## 2 Methods

### 2.1 Participants

The study was approved by the Research Ethics Board at Holland Bloorview Kids Rehabilitation Hospital (ID: 2020-0181-1323, Date: 08-Sep-2020). We recruited 28 typically developed adults. Technical difficulties interrupted data collection for one participant. In total, 27 (N=27) participants, aged 30.2 ± 7.3 years (range: 21–57 years), of whom 25 were right-handed, completed the study. All participants passed a screening to rule out psychological, physiological, neurological conditions or TMS contraindications (Keel et al., 2001;Rossini et al., 2015). Written informed consent was provided by all participants prior to participation.

### 2.2 Equipment

Single TMS pulses were delivered using the Magstim 200^2^stimulator (The Magstim Company Limited, 2005) and a D70 Alpha Coil, with participants seated in a chair featuring a forehead and chin rest to minimize movement. The frameless stereotaxy neuronavigational system (Brainsight, Rogue Research Inc.), equipped with an optical camera and fiducial markers, utilized the MNI (Montreal Neurological Institute) average head model brain for precise coil positioning over the motor region of the participant’s dominant hand. EMG sensors, integrated within the Brainsight system, were placed in a belly-tendon montage on both left and right FDI muscles to monitor MEPs. Video trials were displayed on an approximately 42-inch wall-mounted HP TV, executed by a custom Python script on the Rogue Research TMS mobile computer—a 27” Apple iMac™ with 16 GiB of RAM, and a high-performance graphics processor. Participants were positioned at a comfortable distance in front of the TV.

### 2.3 Experimental session

The participant’s head was co-registered with the MNI average brain template in Brainsight for neuronavigational accuracy. The neuronavigational system was then used to find the motor hotspot and Resting Motor Threshold (RMT) for the dominant hand’s FDI muscle. The coil was navigated through a predefined grid over the primary motor cortex (M1), targeting the area over the central sulcus—home to the “hand knob” area. Grid positioning was tailored to participant handedness, focusing on the hemisphere contralateral to the dominant hand. The coil was oriented at approximately 45 degrees from the midline, tangentially to the scalp, to optimize MEP elicitation in the FDI muscle and identify the strongest MEP-producing grid point as the motor hotspot (Gomez-Tames et al., 2018). The hotspot’s position on the MNI template, coil angle, and orientation were recorded by Brainsight software to guide the TMS pulse delivery throughout the rest of the session. To establish the Resting Motor Threshold (RMT) for the dominant hand’s FDI muscle, we employed a gradual titration method (Rotenberg et al., 2014). This method started with delivering single TMS pulses at subthreshold intensity (~35% of maximum stimulator output; MSO), 5 s apart. Intensity was increased in 5% increments to elicit MEPs ≥50 mV, then finely adjusted by 1% decrements to find the lowest intensity consistently eliciting MEPs above 50 mV in greater than half of ten consecutive pulses.

TMS was delivered at a maximum intensity of 120% RMT to the dominant hand’s motor cortex region in the contralateral hemisphere. TMS-triggered MEPs from the FDI muscles were captured using the integrated EMG system (Rogue Research Inc., Montreal, Canada).

As shown inFigure 1, our spTMS experiments began with two baseline phases. In Baseline 1, participants looked at a white cross on a black background (Fig. 2a) and had 10 MEPs measured at 5-s intervals. Baseline 2 followed a similar protocol but with participants viewing a static image of hands holding stress balls (Fig. 2b).

**Fig. 1.**
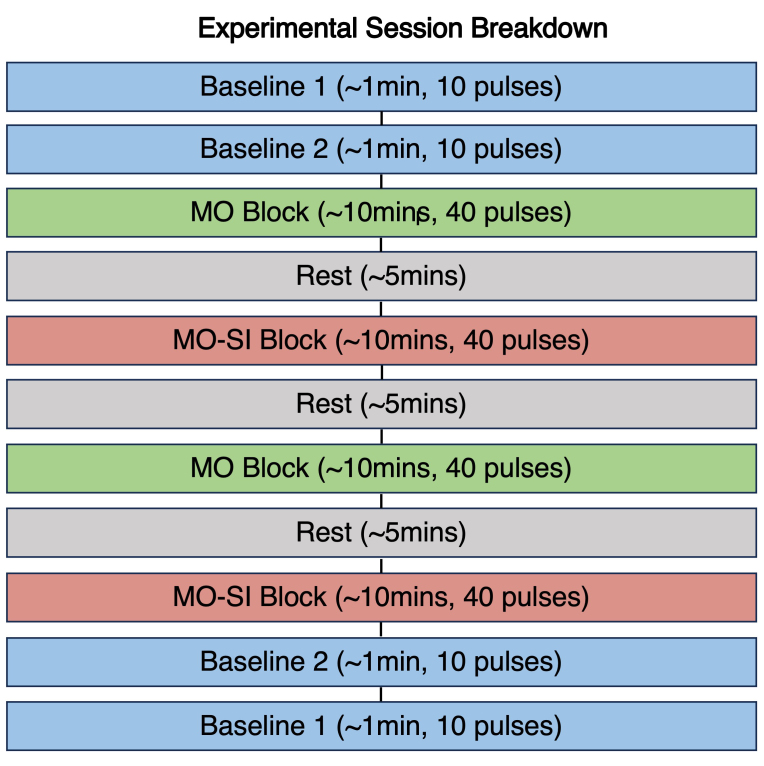
Experimental sequence of the spTMS session comprising baseline (blue), motor observation (green), rest (grey), and motor imitation (red) blocks. MO = motor observation; MO-SI = motor observation with synchronous imitation. Block duration and number of single TMS pulses delivered are shown in parentheses.

**Fig. 2.**
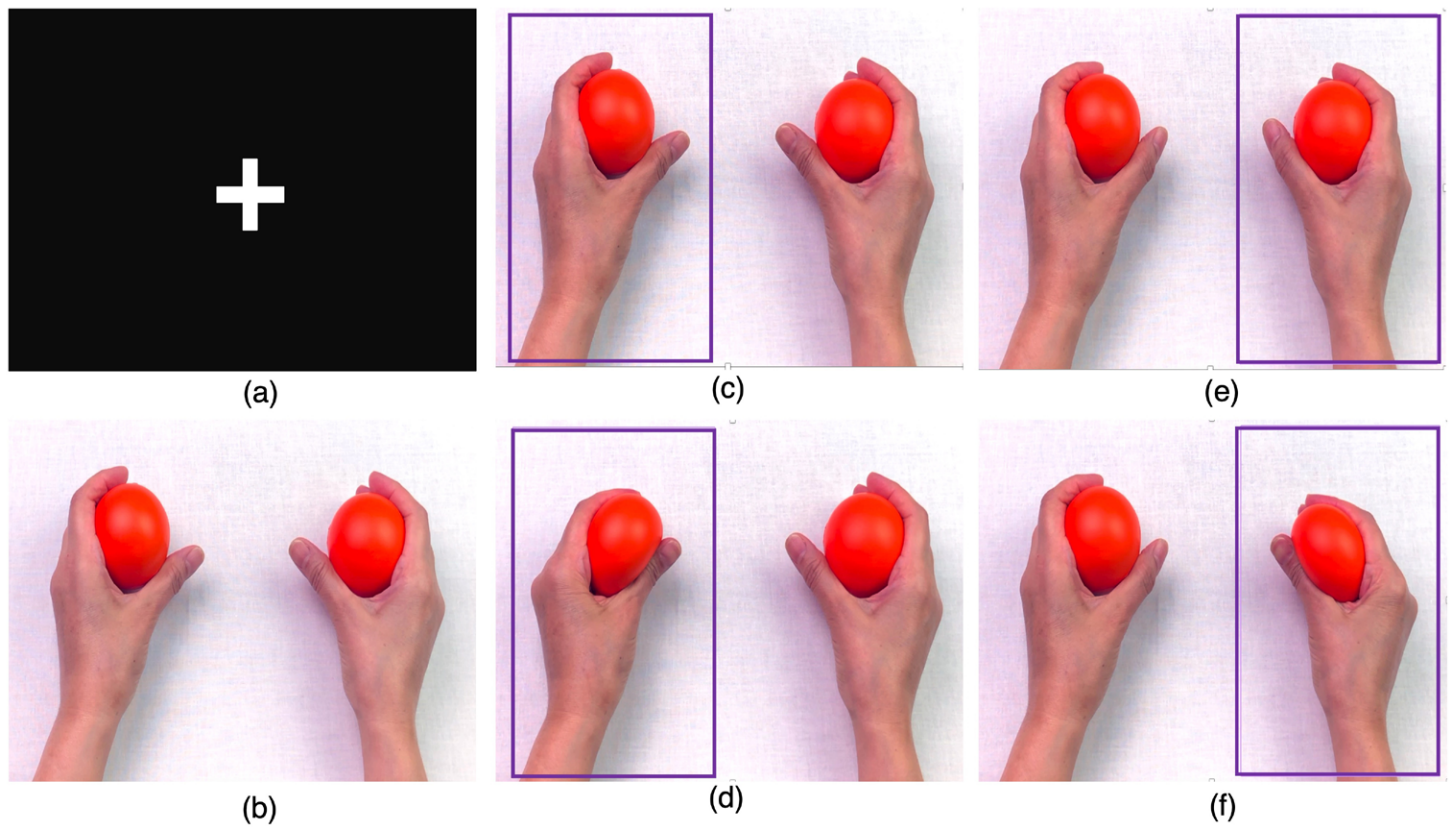
Visual Stimuli and Trial Conditions for spTMS Sessions. (a) Baseline 1; (b) Baseline 2; (c-f) four conditions within the MO and MO-SI blocks: (c) left-hand framed followed by no squeeze; (d) left-hand framed followed by a squeeze; (e) right-hand framed followed by no squeeze, and (f) right-hand framed followed by a squeeze.

The session then advanced into sequences of motor observation (MO) and motor observation with synchronous imitation (MO-SI), each block lasting ten minutes. In the MO blocks, participants viewed videos of a pair of hands from a first-person perspective. They were instructed to passively observe the movements without making any physical movements themselves, remaining still throughout the trials. During these video trials, a visual cue (rectangular frame) enclosed either the left or right hand. This frame functioned as a directive for the participants, guiding their gaze to the specified hand, though it did not unequivocally indicate ensuing hand movement. The framed hand either squeezed the ball or remained stationary. The MO block comprised 40 trials, segmented into four distinct conditions (namely, framing of the left-hand followed by no squeeze, framing of the left-hand followed by a squeeze, framing of the right-hand followed by no squeeze, framing of the right-hand followed by a squeeze;Fig. 2c-f). Each trial lasted for approximately 15 s. Within each trial, one single TMS pulse was administered, either synchronized with the moment of peak squeeze strength or dispersed randomly when the hand remained motionless. In the MO-SI block, participants were instructed to physically imitate the hand movements they observed in the videos in real time. Specifically, when the framed hand squeezed the ball, participants were asked to simultaneously squeeze a ball with their corresponding hand, while remaining still when the hand in the video was stationary. As in the MO block, there were 40 trials, each 15 s in duration, spanning the same four conditions, and following the same TMS pulse delivery protocol.

Rest periods of approximately five minutes were interspersed between blocks. Following the completion of the MO and MO-SI blocks, the session concluded with a replication of Baseline 2 and Baseline 1 blocks. By the end of the session, 10 single TMS pulses were delivered for each baseline condition and 20 TMS pulses for each of the eight experimental conditions (four MO and four MO-SI) per participant.

The four conditions of the MO and MO-SI blocks were pseudo randomly presented. This uncertainty about whether the framed hand would perform an action or remain static, or which hand would be next, enabled the capture of spontaneous neural responses to unexpected motor actions and observations. Participants were instructed to keep their gaze exclusively on the framed hand. Continuous monitoring by the experimenter ensured coil position stability and participant adherence to the visual directives.

### 2.4 Data consolidation

Video trial conditions, originally designated by the side of the framed hand were consolidated across right- and left-handed participants based on hand dominance to facilitate comparative analysis across participants. For example, dominant hand conditions comprised right-hand squeeze and no-squeeze conditions for right-handed participants and the left-hand equivalents for left-handed participants.

### 2.5 Data analysis

Data were analyzed using custom MATLAB scripts. The primary measure was the MEPs’ peak-to-peak amplitude, which reflects corticospinal excitability of the motor pathway during MO and MO-SI conditions. We also measured latency, defined as the interval from the TMS pulse to the first MEP peak (either positive or negative). Latency measures reflect the neural signal conduction velocity, that is, speed and efficiency of neural transmission.

Data from baseline conditions were analyzed separately from experimental conditions for each of MEP peak-to-peak amplitudes and latencies. Baseline datasets were used to investigate any gains in corticospinal excitability accumulated over the session, potentially as a result of repeated stimulations and prolonged exposure to action observation videos, which could confound the results. The experimental condition datasets were used to examine how instructions (MO vs. MO-SI), handedness (dominant vs. non-dominant), and action type (squeeze vs. no squeeze) related to the observed movements influenced MEP peak-to-peak amplitudes and latencies. After sorting measurements within each condition for each participant, the most extreme values beyond 1.5 times the interquartile range (IQR) were removed.

Before statistical analysis, we assessed the normality of the MEP peak-to-peak amplitude and latency datasets using Q-Q plots. Significant deviations from normality were addressed by applying square root, logarithmic, or Box-Cox transformations. The transformation that most normalized the data as judged by Q-Q plots was selected. Since the latency data were concentrated within a narrow range and exhibited minimal variance, no transformations were applied, and the raw latency data were utilized.

Repeated-measures ANOVAs (rmANOVAs) were conducted separately for each dataset. For the baseline datasets, within-subject factors included condition (Baseline 1 and Baseline 2 from pre- and post-experiment phases) and trial (9 trials), forming a 4 x 9 design. For the experimental condition datasets, within-subject factors were instruction type (MO vs. MO-SI), handedness (dominant vs. non-dominant), action type (squeeze vs. no squeeze), and trial (19 trials), resulting in a 2 x 2 x 2 x 19 design. In all analyses, participants were modeled as the between-subject factor to account for inter-individual variability.

The normality of residuals from each repeated-measures model was verified using Q-Q plots. Given the complex factorial design of our study and the potential for sphericity assumption violations, we pre-emptively applied the Greenhouse-Geisser correction to all rmANOVA tests. Significant results (p < 0.05) prompted pairwise post hoc t-tests with Tukey-Kramer correction, and effect sizes were calculated using Cohen’s d.

## 3 Results

The average RMT was 56.15% ± 5.16% MSO (range: 50%–65% MSO). Technical issues led to sporadic measurement losses, requiring the removal of one measurement per condition for each participant to ensure balanced datasets. Outlier removal resulted in a 6% reduction in MEP data. The MEP peak-to-peak amplitude data deviated from normality. The square root transformation yielded the most satisfactory results for achieving normality (Supplementary Fig. 1). Consequently, all MEP amplitude data were transformed using the square root method before further analysis.

### 3.1 Group-level analysis

The rmANOVA results indicated that MEP peak-to-peak amplitudes and latencies from*baseline*conditions (Supplementary Fig. 2) were not significantly different from one another (F (3, 75) = 0.813, Greenhouse-Geisser corrected p (p_GG_) = 0.446,$\eta^{2}=0.031$for MEP amplitudes and F (3, 75) = 0.597, p_GG_= 0.590,$\eta^{2}=0.023$for latencies) and this was consistent among participants (Supplementary Table 1). Hence, modulations in MEP amplitudes and latencies throughout the experiment were not confounded by accumulated gains in corticospinal excitability.

The rmANOVA conducted separately for MEP peak-to-peak amplitudes and latencies of the experimental conditions demonstrated that both measures were significantly influenced by instruction type (amplitudes: F(1, 25) = 42.092, p_GG_< 0.0001, η² = 0.627; latencies: F (1, 25) = 15.489, p_GG_= 0.0006, η² = 0.383), handedness of the observed action (amplitudes: F(1, 25) = 73.775, p_GG_< 0.0001, η² = 0.747; latencies: F (1, 25) = 24.591, p_GG_< 0.0001, η² = 0.496), and type of observed action (amplitudes: F(1, 25) = 58.659, p_GG_< 0.0001, η² = 0.701; latencies: F (1, 25) = 17.294, p_GG_= 0.0003, η² = 0.409), with significant interactions among these factors (Supplementary Table 2). The Q-Q plot of the residuals from all rmANOVAs (on both baseline and experimental conditions) followed a normal distribution (Supplementary Fig. 3).

### 3.2 Post-hoc comparisons

As expected, the MO-SI condition involving the dominant hand squeezing exhibited significantly higher MEP peak-to-peak amplitudes (p << 0.0001) and significantly lower latency (p << 0.0001) compared to all other conditions. This is because the dominant hand’s hemisphere, which is stimulated by single pulse TMS, is also actively engaged in squeezing the ball. Hence, this condition provides reference values of MEP peak-to-peak amplitude and latency.

**Fig. 3.**
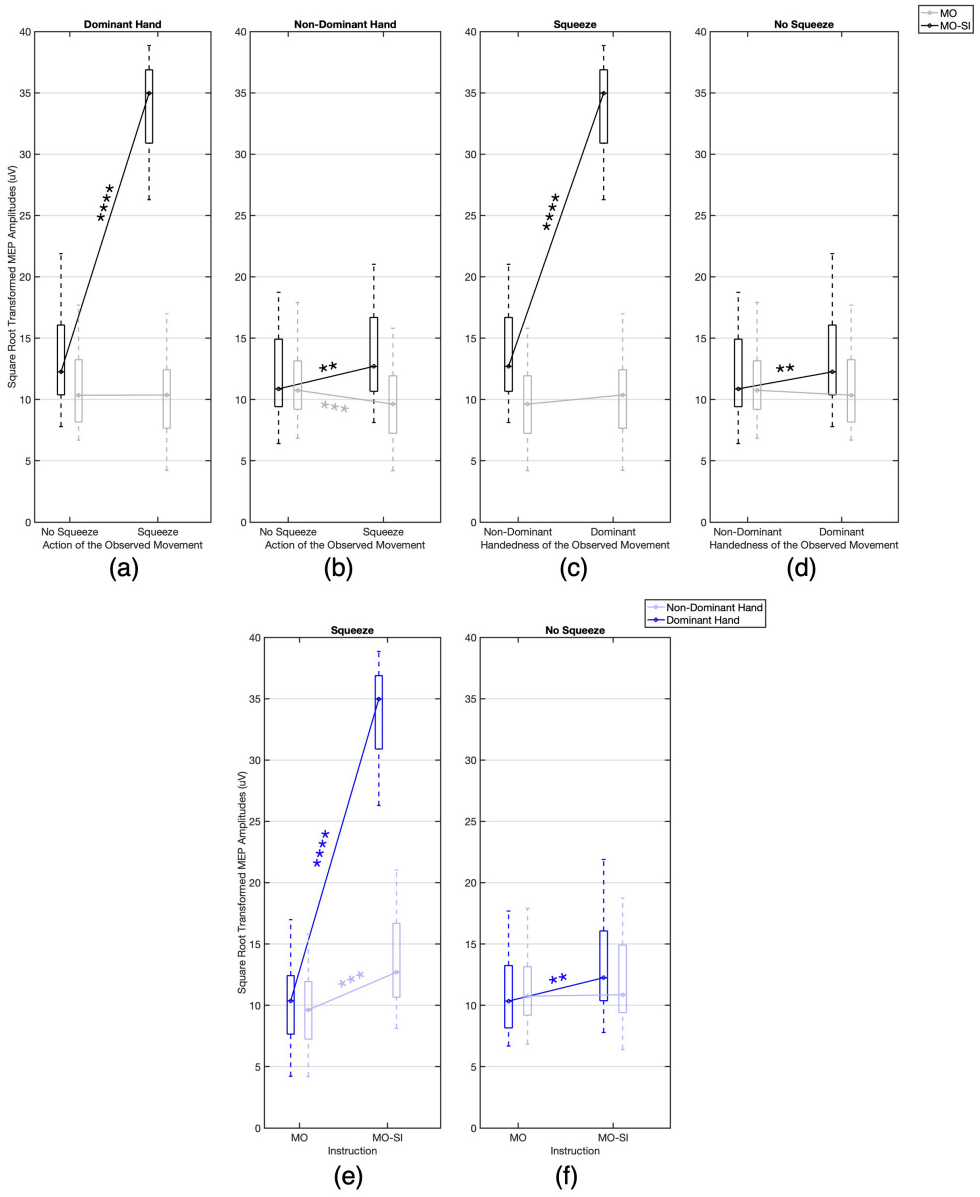
Box plots of square root transformed MEP peak-to-peak amplitudes (uV) across different experimental conditions. Each subplot represents median values and interquartile ranges. Significant differences are marked with asterisks according to their p-values (**p < 0.01, ***p < 0.001, ****p < 0.0001). (a-d) Instruction Effect: comparisons between MO (grey lines) and MO-SI (black lines) instructions when observing dominant and non-dominant hand and for both squeezing and no squeezing actions. (e-f) Handedness Effect: Comparisons between observing non-dominant hand (translucent blue) and dominant hand (blue) for both squeezing and no squeezing actions across MO-SI and MO instructions.

Figure 3a-fillustrate that instruction type, particularly MO-SI, significantly enhanced MEP amplitudes.Figure 3a-bshow, respectively, a pronounced increase in MEP amplitudes during MO-SI of squeezing compared to no squeezing with the dominant (p << 0.0001, Cohen’s d = 6.3381) and non-dominant (p = 0.0038, Cohen’s d = 0.8465) hands. MEP amplitudes were significantly higher in MO-SI of the dominant rather than non-dominant hand whether squeezing (p << 0.0001, Cohen’s d = 5.5147;Fig. 3c) or not squeezing (p = 0.0037, Cohen’s d = 0.8478;Fig. 3d). During MO, a significant change (decrease) in MEP amplitudes only occurred (Fig. 3b) with the non-dominant hand squeezing compared to not squeezing (p << 0.001, Cohen’s d = 1.0900).

Figure 3e-fhighlight the influence of the observed movement’s handedness on MEP amplitudes. MEP amplitudes increased significantly during MO-SI compared to MO, for both dominant (p << 0.0001, Cohen’s d = 5.4254) and non-dominant (p << 0.001, Cohen’s d = 1.1181) hand squeezing (Fig. 3e), but only for the dominant hand when not squeezing (p = 0.0047, Cohen’s d = 0.8293;Fig. 3f).

**Fig. 4.**
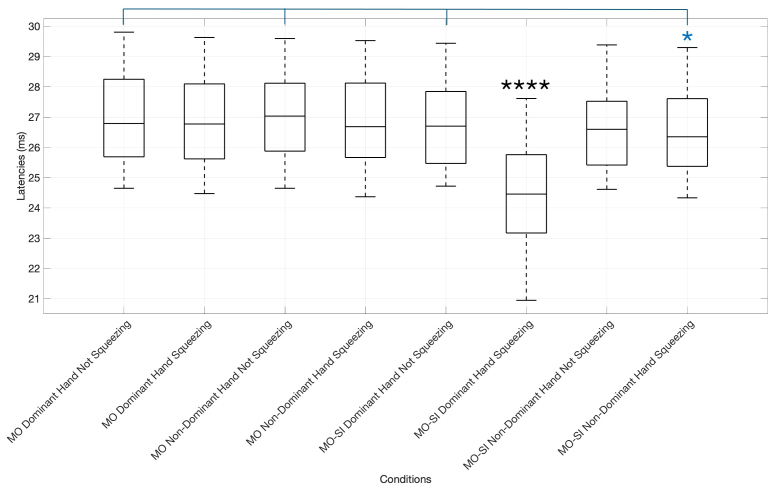
Box plot of MEP latencies across experimental conditions. Significant differences are marked with asterisks according to their p-values (*p < 0.05, ****p < 0.0001). The condition MO-SI Dominant Hand Squeezing exhibited significantly lower MEP latencies compared to all other conditions. Latencies were different between MO-SI Non-Dominant Hand Squeezing and MO Dominant Hand Not Squeezing (p = 0.023), MO Non-Dominant Hand Not Squeezing (p = 0.031), and MO-SI Dominant Hand Not Squeezing (p = 0.030).

Figure 4portrays the differences in MEP latencies among the various conditions, emphasizing the pronounced reduction in latencies in the synchronous imitation of dominant hand squeezing condition and the significantly lower latencies in the synchronous imitation of non-dominant hand squeezing compared to three other not squeezing conditions.

## 4 Discussion

Our study utilized spTMS to explore the MNS’s response to observing and imitating dominant and non-dominant hand movements coordinated by lateralized cues. Our results suggest that motor imitation, or the intention to imitate, broadly enhances corticospinal excitability across varied conditions. This effect underscores the mirror neuron system’s sensitivity to direct engagement in observed actions.

### 4.1 Observation with motor intention enhances MNS activation

We found that the mere intention to imitate, but without actual movement, significantly enhances neural excitability. This finding highlights the brain’s sensitivity to movement intention: the anticipation of motor imitation can evoke measurable changes in neural excitability. It seems that cognitive engagement with the task, such as planning or intending to mimic observed actions primes the motor system for activation. This priming, in turn, reflects the MNS’s capacity to integrate sensory and motor information. Conversely, passive observation of hand actions without motor intention, regardless of whether the actions involved a dominant hand squeeze, no squeeze, or non-dominant hand actions, did not significantly enhance corticospinal excitability. It thus appears that the brain preferentially stimulates neural pathways critical for motor control and learning during observation yoked with motor intention.

This finding is consistent with that ofMcGregor et al. (2018)who reported that observing motor learning of an upper extremity reaching task in a robot-applied force field significantly increased MEP amplitudes, whereas observation of an unlearnable randomly varying force field did not. In other words, goal-directed learning activated the MNS beyond that of mere passive observation. Similarly,de Beukelaar et al. (2016)found that the observation of whole hand or precision grasps when preceded by a pre-cue revealing the type of upcoming grasp led to early increases in MEPs. These studies support the notion that activation of the MNS is enhanced when observers are not just passively watching but actively engaged with the actions being observed.

Our findings appear to contradict those ofHardwick et al. (2012)who reported a facilitatory effect on MEPs only during observation only (what they term as observe-to-attend) trials, but not during observation with an intention to imitate (observe-to-imitate) trials. They rationalized that there may be an inhibitory mechanism that modulates corticospinal excitability in response to intention to imitate. However, Hardwick et al. cued participants to observe first, and subsequently, to imitate whereas our protocol encouraged spontaneous imitation with the observed action. Hence, the speculated inhibitory response may have arisen to suppress immediate overt imitation. Our spontaneous imitation protocol likely taps into a more direct mirroring process, as evidenced by the increased corticospinal excitability in our observe with synchronous imitation trials. The contrast between our study and Hardwick et al. suggests that both the timing of imitation and the observer’s intention, are critical in shaping the neural outcomes of action observation.

Passive observation of an action alone did not significantly enhance corticospinal excitability in our study. In fact, we found a significant inhibition in corticospinal excitability when participants observed the squeezing action of the non-dominant hand.Borroni and Baldissera (2008)reported enhanced motor pathway excitability when participants observed a ball grasping task without motor intention. This discrepancy with our findings may be attributed to the fact that Borroni and Baldissera presented video clips of cyclic up-and-down oscillation of the hand from an allocentric (third person) perspective whereas our videos adopted an egocentric perspective. The allocentric perspective might engage the MNS more effectively by mirroring social interactions where the system is typically activated, implying that observer perspective is important in eliciting motor resonance.Prinsen et al. (2017)andPrinsen & Alaerts (2020)showed that direct eye contact with the performer during observation of an action significantly boosts corticospinal excitability. Indeed, socially contextualized, complex actions can evoke strong activation of the MNS (Stolbkov & Gerasimenko, 2021). Our larger participant pool (27 participants) compared to Borroni & Baldissera (5 participants) may have also introduced greater variability in neural responses. Additionally, none of these studies have examined corticospinal excitability during the observation of the non-dominant hand adjacent to the dominant hand. The significant inhibition we observed during the squeezing action of the non-dominant hand could be attributed to the competitive interaction between the hemispheres or the lack of motor dominance associated with the non-dominant hand. Further research is needed to explore these mechanisms.

### 4.2 Hemispheric dominance

We found a marked increase in corticospinal excitability to the dominant hand during imitation tasks involving the non-dominant hand. This effect reveals a robust engagement of the dominant hemisphere (ipsilateral to the non-dominant hand, notably the left hemisphere for 92% of participants given that 25 out of 27 were right-handed), as assessed by spTMS.Tian et al. (2021)studied the neural responses to action observation and imitation through the lens of M1–M1 interhemispheric inhibition amidst varying hand dominance and imitation patterns. They reported activation of the motor cortex ipsilateral to the movement being imitated, consistent with our observations of increased excitability in the dominant hemisphere during tasks involving the non-dominant hand. This finding points to the prevailing role of the dominant hemisphere in integrating observed and executed actions, irrespective of the laterality of motor observation or imitation (Serrien et al., 2003).

### 4.3 Insights into MNS

Our finding that imitation with the non-dominant hand enhanced corticospinal excitability in the dominant hemisphere spotlights the MNS’s inherent bilateral nature. This bilaterality encompasses the critical interconnectedness between the precentral gyrus, inferior parietal lobule, and the superior temporal sulcus, and accentuates their cooperative role in processing and replicating observed actions (Cerri et al., 2015). The necessity of cognitive engagement for effective mirror neuron activation implicates the posterior part of the inferior frontal gyrus and the adjacent sector of the ventral premotor cortex (Iacoboni et al., 2005). The reliance of the MNS on higher-order cognitive processes appears vital for transforming observed actions into executable motor commands. Lastly, our results imply proactive engagement of frontal and parietal regions in readying the motor system for action execution. In concert with the primary motor cortex, these areas are instrumental in executing motor commands (Rizzolatti & Sinigaglia, 2010) and underscore the capacity of the MNS for instant action imitation.

### 4.4 Clinical implications

The finding that motor intention in observation is required for significant motor neuronal system activation may inform optimization of rehabilitation approaches that rely on motor resonance to invoke neural plasticity. One such rehabilitation approach is brain state-dependent brain stimulation (BSDBS), which uses brain-computer interfaces (BCIs) to classify brain states in real time obtained through brain monitoring techniques, especially those with high temporal resolution such as electroencephalography (EEG) and electrocorticography (ECoG). Brain stimulation is then delivered at moments when the brain is optimally primed for neuroplastic changes, enhancing the effectiveness of rehabilitation and therapeutic interventions. For example, the optimal brain state for motor rehabilitation, typically defined as sensorimotor event-related desynchronization (ERD), is often induced through motor imagery. Given the difficulties in performing motor imagery effectively post-injury, in pediatric cases, or due to cognitive impairments, studies have explored passive movement observation as an alternative (Spruijt et al., 2015;Zhang et al., 2024). In concert with these studies, our findings contend that passive observation can induce ERD but further stipulate the necessity of cognitive engagement for enhancement of corticospinal excitability. Future BSDBS protocols, especially for patients with limited capacity for motor imagery, could thus benefit from incorporating strategies to enhance cognitive engagement during motor observation, potentially through interactive or immersive experiences.

### 4.5 Limitations

There were several limitations in our study. Despite using a headrest, chin rest, and stationary arm for precise TMS probe positioning, completely restricting participant movement was challenging, leading to occasional deviations in TMS targeting. However, we frequently readjusted the TMS probe and utilized the neuronavigation system to correct positional inaccuracies in real-time. A larger study sample would enhance statistical power and the detection of subtler effects. Additionally, administering 20 TMS pulses per condition, which minimized session duration, may have potentially limited the depth of insights into TMS-evoked EMG responses. Future studies could benefit from increasing the pulse number.

Our study sample included two left-handed individuals. Although re-analysis of our data excluding left-handed participants did not significantly change our findings, the issue of hemispheric dominance deserves closer examination. For example,Tian et al. (2021)reported asymmetric interhemispheric inhibition (IHI) in right-handed individuals and more balanced IHI in left-handed individuals. Future research may aim to recruit a balance of left-handed individuals to disaggregate findings on corticospinal excitability by dominant hemisphere.

While our study adheres to the methodological standards of state-dependent TMS research, where TMS alone is often used to measure how task-related activity influences corticospinal excitability via MEPs, our MEP data are limited in its capacity to probe the full progression of brain dynamics during our experimental tasks. Future research in action observation and imitation protocols will benefit from implementing TMS-EEG protocols instead of TMS alone. TMS-EEG protocols allow researchers to track brain responses to TMS not only at the peripheral level (through MEPs) but also directly at the cortical level, simultaneously measuring brain dynamics in real time (Hernandez-Pavon et al., 2023;Tremblay et al., 2019). EEG could provide valuable information on earlier neural processes, capturing brain activity during the preparatory and anticipatory phases of movement observation or imitation, as well as cortical excitability that does not reach the threshold for activation detectable by MEPs. TMS-EEG also enables the exploration of both immediate TMS-evoked potentials (TEPs) and longer-lasting changes in brain oscillatory activity following stimulation, offering deeper insights into how cortical networks respond to action observation and imitation.

## 5 Conclusion

We studied neurophysiological responses elicited by hand movement observation and imitation, employing spTMS in 27 typically developed adults. Our findings elucidate the necessity of cognitive engagement (motor intentionality) for the enhancement of corticospinal excitability and the presence of bilateral hemispheric activation in unilateral action observation and imitation. Importantly, passive observation, in the absence of active cognitive engagement, failed to significantly modulate corticospinal excitability. Our findings may inform the optimization of rehabilitation strategies leveraging observation therapy and BSDBS protocols to maximize functional recovery through effective MNS activation.

## Supplementary Material

Supplementary Material

## Data Availability

The datasets analyzed and analysis code for the current study are available from the corresponding author on reasonable request.
