# Supplementary material for: Action observation and imitation: Corticospinal responses and hemispheric dominance
Source: Imaging Neurosci (Camb). 2025 Jan 3;3:imag_a_00419. doi: 10.1162/imag_a_00419 (PMC12319879; doi:10.1162/imag_a_00419)
Supplement: Supplementary Material [file imag_a_00419-supp.pdf]

Action Observation and Imitation:
Corticospinal Responses and Hemispheric Dominance
(Supplementary Document)

Niloufaralsadat Hashemi[1,2*], Tom Chau[1,2†], and Deryk Beal [1,3‡]

[1] *Bloorview Research Institute, Holland Bloorview Kid's Rehabilitation Hospital, Toronto, Ontario, Canada, M4G 1R8*
[2] *Institute of Biomedical Engineering, University of Toronto, Toronto, Ontario, Canada, M5S 3G9*
[3] *Speech-Language Pathology, University of Toronto, Toronto, Ontario, Canada, M5G 1V7*

*E-mail: nhashemi@hollandbloorview.ca
†E-mail: tchau@hollandbloorview.ca
‡E-mail: dbeal@hollandbloorview.ca (***corresponding author***)

Data Transformations

The MEP peak-to-peak amplitude data deviated from normality, as indicated by Q-Q plot inspections. Among the transformations tested (i.e., square root, logarithmic, or Box-Cox), the square root transformation yielded the most satisfactory results in terms of aligning with the normality assumption. Consequently, all MEP amplitude data were transformed using the square root method before further analysis. The Q-Q plot of the square root transformed MEP amplitudes are shown in Figure 1.

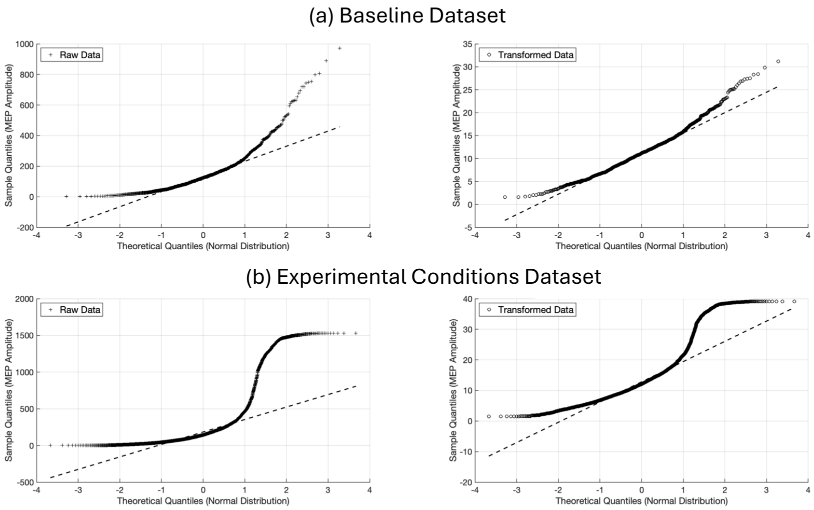

Figure 1 - Q-Q plots of raw and square root transformed MEP peak-to-peak amplitude data: (a) baseline dataset, (b) experimental condition dataset. The left subplots show the raw data with '+' markers and the right subplots show the transformed data with 'o' markers. The reference lines are depicted as dashed lines.

Group-Level Analysis

The rmANOVA results indicated that MEP peak-to-peak amplitudes and latencies from baseline conditions were not significantly different from one another. Figure 2 presents the box plot of the MEP peak-to-peak amplitude and latency measurements across the baseline conditions, providing a visual representation of the data distribution.

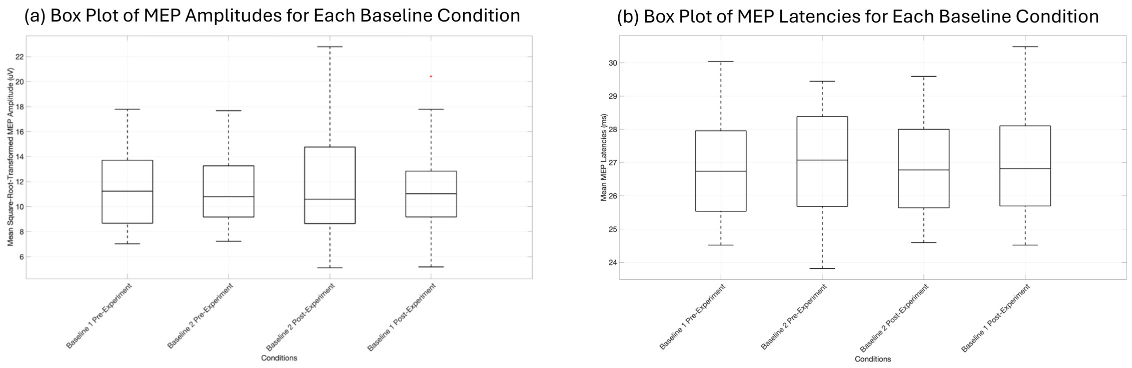

Figure 2 - The box plots display the distribution of mean MEP peak-to-peak amplitudes (us) (a) and latencies (ms) (b) for each baseline condition. The plot provides a visual representation of the data distribution across the baseline conditions, showing no significant differences in MEP peak-to-peak amplitudes or latencies between the conditions.

Repeated measures ANOVAs (rmANOVAs) were conducted separately for each baseline datasets. Within-subject factors included baseline conditions (baseline 1 and baseline 2 from pre- and post-experiment phases) and trial (9 trials), forming a 4x9 design. Subjects were modeled as the between-subject variable to account for any inter-individual variability. The rmANOVA results are summarized in Table 1.

Table 1 - Summary of rmANOVA results for MEP peak-to-peak amplitudes and latencies across baseline conditions. This table provides a comprehensive summary of the statistical analyses conducted. 'Subject' is designated as a between-subject variable, whereas 'Condition' and 'Trial' are designated as within-subject variables. Included in the table are F-values, p-values, and effect sizes (η^2) for each analysis.

**Statistical Analysis**	**MEP peak-to-peak Amplitudes**	**MEP Latencies**
rmANOVA (Subject)	$F(1, 25) = 8.199$, $p_{GG} = 0.008$, $\eta^2 = 0.247$	$F(1, 25) = 0.225$, $p_{GG} = 0.640$, $\eta^2 = 0.009$
rmANOVA Condition	$F(3, 75) = 0.813$, $p_{GG} = 0.446$, $\eta^2 = 0.031$	$F(3, 75) = 0.597$, $p_{GG} = 0.590$, $\eta^2 = 0.023$
rmANOVA (Subject): Condition	$F(3, 75) = 0.664$, $p_{GG} = 0.515$, $\eta^2 = 0.026$	$F(3, 75) = 0.963$, $p_{GG} = 0.403$, $\eta^2 = 0.037$
rmANOVA Trial	$F(1, 25) = 202.21$, $p_{GG} < 0.001$, $\eta^2 = 0.890$	$F(1, 25) = 1867.8$, $p_{GG} < 0.001$, $\eta^2 = 0.987$
rmANOVA (Subject): Trial	$F(1, 25) = 7.387$, $p_{GG} = 0.012$, $\eta^2 = 0.228$	$F(1, 25) = 0.496$, $p_{GG} = 0.488$, $\eta^2 = 0.019$
rmANOVA Condition x Trial	$F(3, 75) = 0.844$, $p_{GG} = 0.434$, $\eta^2 = 0.033$	$F(3, 75) = 0.677$, $p_{GG} = 0.517$, $\eta^2 = 0.026$
rmANOVA (Subject): Condition x Trial	$F(3, 75) = 0.530$, $p_{GG} = 0.588$, $\eta^2 = 0.033$	$F(3, 75) = 1.129$, $p_{GG} = 0.332$, $\eta^2 = 0.043$

Repeated measures ANOVAs (rmANOVAs) were also conducted separately for each experimental condition datasets. within-subject factors were instruction type (MO vs. MO-SI), handedness of the observed action (dominant vs. non-dominant), action type of the observed action (squeeze vs. no squeeze), and trial (19 trials), resulting in a 2x2x2x19 design. Subjects were modeled as the within-subject variable to account for inter-individual variability. The rmANOVA results are summarized in Table 2.

Table 2 - Summary of rmANOVA results for MEP peak-to-peak amplitudes and latencies from experimental conditions. Within-subject variables include 'Instruction', 'Handedness', and 'Action'. 'Subject' is modeled as the between-subject variable.

**Statistical Analysis**	**MEP peak-to-peak Amplitudes**	**MEP Latencies**
rmANOVA (Subject)	$F(1, 25) = 8.343$, $p_{GG} = 0.0079$, $\eta^2 = 0.250$	$F(1, 25) = 0.148$, $p_{GG} = 0.704$, $\eta^2 = 0.006$
rmANOVA Instruction	$F(1, 25) = 42.092$, $p_{GG} < 0.0001$, $\eta^2 = 0.627$	$F(1, 25) = 15.489$, $p_{GG} = 0.0006$, $\eta^2 = 0.383$
rmANOVA (Subject): Instruction	$F(1, 25) = 0.624$, $p_{GG} = 0.437$, $\eta^2 = 0.0243$	$F(1, 25) = 0.267$, $p_{GG} = 0.609$, $\eta^2 = 0.0106$

rmANOVA<br>Handedness	F (1, 25) = 73.775, $p_{GG} < 0.0001$, $\eta^2 = 0.747$	F (1, 25) = 24.591, $p_{GG} < 0.0001$, $\eta^2 = 0.496$
rmANOVA<br>(Subject): Handedness	F (1, 25) = 1.478, $p_{GG} = 0.235$, $\eta^2 = 0.0558$	F (1, 25) = 0.508, $p_{GG} = 0.483$, $\eta^2 = 0.0199$
rmANOVA<br>Action	F (1, 25) = 58.659, $p_{GG} < 0.0001$, $\eta^2 = 0.701$	F (1, 25) = 17.294, $p_{GG} = 0.0003$, $\eta^2 = 0.409$
rmANOVA<br>(Subject): Action	F (1, 25) = 0.176, $p_{GG} = 0.679$, $\eta^2 = 0.007$	F (1, 25) = 0.184, $p_{GG} = 0.671$, $\eta^2 = 0.007$
rmANOVA<br>Trial	F (1, 25) = 329.22, $p_{GG} < 0.001$, $\eta^2 = 0.929$	F (1, 25) = 2020.5, $p_{GG} < 0.001$, $\eta^2 = 0.987$
rmANOVA<br>(Subject): Trial	F (1, 25) = 8.401, $p_{GG} = 0.0077$, $\eta^2 = 0.251$	F (1, 25) = 0.317, $p_{GG} = 0.578$, $\eta^2 = 0.0125$
rmANOVA<br>Instruction x Trial	F (1, 25) = 35.724, $p_{GG} < 0.0001$, $\eta^2 = 0.588$	F (1, 25) = 12.092, $p_{GG} = 0.0019$, $\eta^2 = 0.326$
rmANOVA<br>(Subject): Instruction x Trial	F (1, 25) = 0.707, $p_{GG} = 0.408$, $\eta^2 = 0.0275$	F (1, 25) = 0.662, $p_{GG} = 0.424$, $\eta^2 = 0.0258$
rmANOVA<br>Handedness x Trial	F (1, 25) = 65.038, $p_{GG} < 0.0001$, $\eta^2 = 0.722$	F (1, 25) = 14.33, $p_{GG} = 0.0009$, $\eta^2 = 0.364$
rmANOVA<br>(Subject): Handedness x Trial	F (1, 25) = 1.32, $p_{GG} = 0.261$, $\eta^2 = 0.0501$	F (1, 25) = 0.151, $p_{GG} = 0.701$, $\eta^2 = 0.006$
rmANOVA<br>Action x Trial	F (1, 25) = 51.364, $p_{GG} < 0.0001$, $\eta^2 = 0.672$	F (1, 25) = 9.306, $p_{GG} = 0.0053$, $\eta^2 = 0.271$
rmANOVA<br>(Subject): Action x Trial	F (1, 25) = 0.204, $p_{GG} = 0.655$, $\eta^2 = 0.0081$	F (1, 25) = 0.226, $p_{GG} = 0.639$, $\eta^2 = 0.009$
rmANOVA<br>Instruction x Handedness	F (1, 25) = 90.232, $p_{GG} < 0.0001$, $\eta^2 = 0.783$	F (1, 25) = 11.181, $p_{GG} = 0.0026$, $\eta^2 = 0.309$
rmANOVA<br>(Subject): Instruction x Handedness	F (1, 25) = 2.507, $p_{GG} = 0.126$, $\eta^2 = 0.0911$	F (1, 25) = 0.00163, $p_{GG} = 0.968$, $\eta^2 = 0.0007$
rmANOVA<br>Instruction x Action	F (1, 25) = 163.61, $p_{GG} < 0.0001$, $\eta^2 = 0.867$	F (1, 25) = 18.772, $p_{GG} = 0.0002$, $\eta^2 = 0.429$
rmANOVA<br>(Subject): Instruction x Action	F (1, 25) = 0.131, $p_{GG} = 0.720$, $\eta^2 = 0.0052$	F (1, 25) = 0.653, $p_{GG} = 0.427$, $\eta^2 = 0.025$
rmANOVA<br>Handedness x Action	F (1, 25) = 160.59, $p_{GG} < 0.0001$, $\eta^2 = 0.865$	F (1, 25) = 18.796, $p_{GG} = 0.0002$, $\eta^2 = 0.429$
rmANOVA<br>(Subject): Handedness x Action	F (1, 25) = 2.724, $p_{GG} = 0.111$, $\eta^2 = 0.098$	F (1, 25) = 0.170, $p_{GG} = 0.684$, $\eta^2 = 0.0067$
rmANOVA<br>Instruction x Handedness x Trial	F (1, 25) = 92.098, $p_{GG} < 0.0001$, $\eta^2 = 0.786$	F (1, 25) = 8.476, $p_{GG} = 0.0075$, $\eta^2 = 0.253$
rmANOVA<br>(Subject): Instruction x Handedness x Trial	F (1, 25) = 3.339, $p_{GG} = 0.080$, $\eta^2 = 0.1178$	F (1, 25) = 0.00246, $p_{GG} = 0.961$, $\eta^2 = 0.0001$
rmANOVA<br>Instruction x Action x Trial	F (1, 25) = 170.26, $p_{GG} < 0.0001$, $\eta^2 = 0.872$	F (1, 25) = 17.28, $p_{GG} = 0.0003$, $\eta^2 = 0.409$
rmANOVA<br>(Subject): Instruction x Action x Trial	F (1, 25) = 0.295, $p_{GG} = 0.592$, $\eta^2 = 0.0117$	F (1, 25) = 0.513, $p_{GG} = 0.480$, $\eta^2 = 0.020$
rmANOVA<br>Handedness x Action x Trial	F (1, 25) = 89.975, $p_{GG} < 0.0001$, $\eta^2 = 0.783$	F (1, 25) = 14.519, $p_{GG} = 0.0008$, $\eta^2 = 0.367$
rmANOVA<br>(Subject): Handedness x Action x Trial	F (1, 25) = 3.049, $p_{GG} = 0.093$, $\eta^2 = 0.109$	F (1, 25) = 0.00065, $p_{GG} = 0.980$, $\eta^2 = 0.00003$
rmANOVA<br>Instruction x Handedness x Action	F (1, 25) = 98.209, $p_{GG} < 0.0001$, $\eta^2 = 0.797$	F (1, 25) = 14.67, $p_{GG} = 0.0008$, $\eta^2 = 0.370$
rmANOVA<br>(Subject): Instruction x Handedness x Action	F (1, 25) = 4.715, $p_{GG} = 0.040$, $\eta^2 = 0.159$	F (1, 25) = 0.00006, $p_{GG} = 0.994$, $\eta^2 = 2.564e-6$

rmANOVA Instruction x Handedness x Action x Trial	$F\ (1, 25) = 62.334$, $p_{GG} < 0.0001$, $\eta^2 = 0.714$	$F\ (1, 25) = 12.41$, $p_{GG} = 0.0017$, $\eta^2 = 0.332$
rmANOVA (Subject): Instruction x Handedness x Action x Trial	$F\ (1, 25) = 4.572$, $p_{GG} = 0.042$, $\eta^2 = 0.155$	$F\ (1, 25) = 0.033$, $p_{GG} = 0.857$, $\eta^2 = 0.0013$

The normality of residuals from each repeated measures model was verified using Q-Q plots to ensure adherence to ANOVA assumptions. These Q-Q plots are shown in Figure 3.

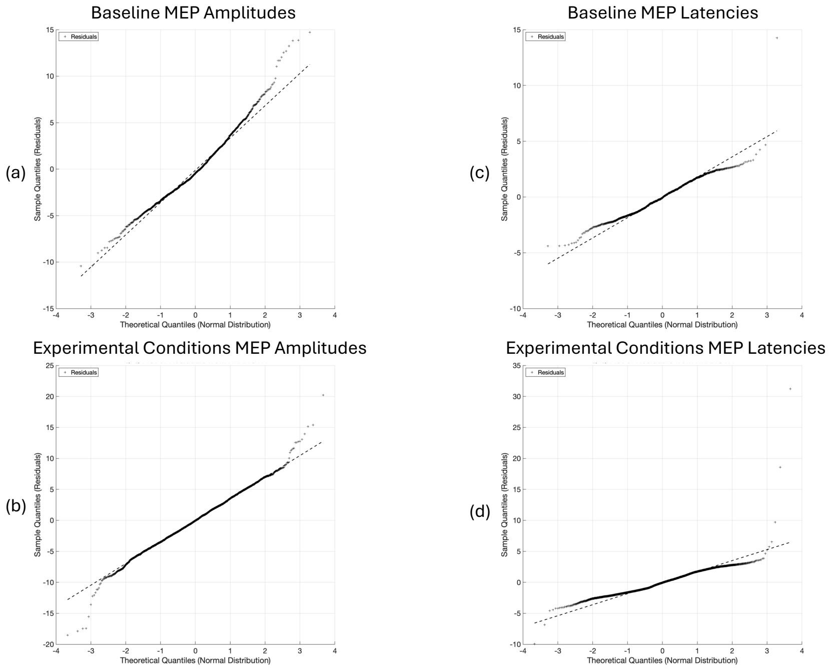

Figure 3 - Q-Q plots of the residuals from the rmANOVA models for the MEP amplitudes baseline (a) and experimental condition (b) datasets and for the MEP latencies baseline (c) and experimental condition (d) datasets.
